# Meteorin-like/Metrnl, a novel secreted protein implicated in inflammation, immunology, and metabolism: A comprehensive review of preclinical and clinical studies

**DOI:** 10.3389/fimmu.2023.1098570

**Published:** 2023-02-24

**Authors:** Zhuoqi Li, Ziyu Gao, Tao Sun, Shipeng Zhang, Shengnan Yang, Meilin Zheng, Hui Shen

**Affiliations:** ^1^ Department of Rheumatology and Immunology, The First Hospital of China Medical University, China Medical University, Shen Yang, China; ^2^ Department of Thyroid Surgery, The First Hospital of China Medical University, China Medical University, Shen Yang, China

**Keywords:** Metrnl, inflammation, metabolism, immunity, bone, heart, obesity, diabetes

## Abstract

Meteorin-like, also known as Metrnl, Meteorin-β, Subfatin, and Cometin, is a novel secreted protein exerting pleiotropic effects on inflammation, immunology, and metabolism. Earlier research on this hormone focused on regulating energy expenditure and glucose homeostasis. Consequently, several studies attempted to characterize the molecule mechanism of Metrnl in glucose metabolism and obesity-related disorders but reported contradictory clinical results. Recent studies gradually noticed its multiple protective functions in inflammatory immune regulations and cardiometabolic diseases, such as inducing macrophage activation, angiogenesis, tissue remodeling, bone formation, and preventing dyslipidemias. A comprehensive understanding of this novel protein is essential to identify its significance as a potential therapeutic drug or a biomarker of certain diseases. In this review, we present the current knowledge on the physiology of Metrnl and its roles in inflammation, immunology, and metabolism, including animal/cell interventional preclinical studies and human clinical studies. We also describe controversies regarding the data of circulation Metrnl in different disease states to determine its clinical application better.

## Introduction

Secreted proteins play an important role in certain physiological or pathological processes to reflect and regulate the organism’s state at the molecular level. Exploring the secreted protein’s mechanism, which may be applied as a targeted agent or a predictive biomarker, would help diagnose and treat clinical diseases. Metrnl, also known as Meteorin-like, Meteorin-β, Subfatin, and Cometin, is a novel secretory protein that shares 46% amino acid sequence homology with a neurotrophic factor named Meteorin. Unlike Meteorin’s brain-specific expression in the central nervous system, Metrnl is abundant in metabolism-related organs and barrier tissues (1). Besides, its secretion and regulation depend not on the fixed model of cell types but on the concrete physiological and pathological context.

When Metrnl was described in early studies as an identity of adipokine, exerting pleiotropic effects on glucose homeostasis, such as improving insulin sensitivity, facilitating adipose tissue browning, and increasing energy expenditure (2). While recent research results suggested that Metrnl may play protective roles in several cardio-metabolic and other inflammatory immune diseases. Different physiological activities could also regulate Metrnl expression, including exercise, temperature variation, bariatric surgery, and high-fat diets. A comprehensive understanding of Metrnl is essential to determine its significance as a potential therapeutic agent or biomarker for certain diseases. As such, this present review would offer an overview of the valid evidence on the effects of Metrnl in inflammation, immunology, metabolism, and related diseases, including animal/cell interventional preclinical studies and human clinical studies.

## Physiology of Metrnl

Metrnl is located on regions of human chromosome 17q25.3 and mouse chromosome 11qE2, consisting of 311 amino acids encoded by 936 base pairs (3). There is a 45 amino acid sequence at the amino terminus, but Metrnl does not have a membrane-spanning region. Since Metrnl was the only gene on the q-arm terminal end of chromosome 17, it may regulate a mild clinical phenotype of a rare disorder named Mild Ring 17 Syndrome (4). This phenotype presented as growth delay and intellectual disability and was presumed to be associated with abnormal expression of genes (including Metrnl) related to proximal telomere side neurogenesis. Interestingly, 17q25.3 was a susceptibility locus of cardiovascular disease (5), psoriasis (6), and atopic dermatitis (7), where its fragment deletion could result in cardiovascular deficiencies or cardiac phenotype changes. Notably, Metrnl has been studied preliminarily in these diseases, thus providing a genomic rationale for involvement in pathogenesis development. However, its intracellular localization and distribution still need to be clarified. Its molecular structure and family ligands are also poorly studied, which poses an obstacle to exploring the relationship between Metrnl structure and function.

Metrnl was highly conservative in its evolution and 40% homologous to the neurotrophic factor Meteorin (8). Unlike Meteorin’s concentrated expression in the central nervous system (CNS), Metrnl was highly expressed in adipose, skin, and mucosal barrier tissue, whereas less in CNS (1, 9). Nevertheless, several studies still attempted to explore Metrnl functions in CNS. Based on original bioinformatics, Ramialison et al. (10) detected Metrnl as one of the direct downstream targets of PAX2/5/8 genes involved in inner ear growth. A similar study was reported by Jørgensen et al. (3) that Metrnl exclusively in dorsal root ganglions and inner ear during early mouse development but not in the adult CNS. These studies suggest Metrnl may be involved in inner ear development.

As a new neurotrophic factor, Metrnl could promote neurite outgrowth, migration, and neuroprotection (3, 11). Of note, Metrnl was able to cross the human blood-brain barrier to migrate into the CNS, as well as Metrnl levels in human cerebrospinal fluid were significantly correlated with its serum levels and albumin CSF/serum ratios (12). Considering Metrnl could induce significantly upon exercise and muscle contraction, prompting that Metrnl may migrate from the periphery tissues into the CNS under these activities. However, to address the effect of *de novo* innervation on human skeletal muscle cells, Jan et al. (13) found that co-culture myotubes with the embryonic rat spinal cord explants did not affect Metrnl mRNA expression. It may be interesting to explore whether Metrnl directly influences the muscle-brain axis or whether Metrnl involves in neuromuscular transmission. Recently, Hong et al. (14) reported that Metrnl could regulate cognitive dysfunction in D-galactose-induced aging models as Metrnl deficiency significantly aggravated the cognitive impairment and decreased hippocampal BDNF, TrkB, and GFAP levels. It would be promising that exploring Metrnl as a candidate treatment and alleviation of aging-related cognitive dysfunction. But further studies are required to clarify the specific mechanisms among Metrnl, neurons and other glial cells.

## Metrnl and immunology

Considering Metrnl is involved in modulating the immune response, several studies have explored its implications in abnormal immune conditions, detected the expression of Metrnl in autoimmune diseases, and analyzed its relationship with these diseases. In this part, we described current knowledge of Metrnl’s role in immunology, including regulations of innate or adaptive immunity and involvement in autoimmune diseases. We also summarized main findings of Metrnl-related preclinical (**Table 1**) and clinical studies (**Table 2**) on immunology to understand better.

**Table 1:** Summary of preclinical studies associated with Metrnl in inflammation and immunology.

Year	Ref.	Methods	Main findings	
			Immunology	Inflammation
2014	(15)	Mouse; *in vitro*	Trigger Type 2 immune cascade activation, promote M2 alternative activation of macrophages	Increase anti-inflammatory cytokines
2015	(9)	*In vitro*	Produced by M2-like macrophages	Overexpression in skin lesions of skin inflammatory diseases
2016	(16)	Mouse	Intestinal epithelial cells Metrnl-KO mouse: decrease gut antimicrobial peptides	–
2018	(17)	Mouse; *in vitro*	Metrnl^−/−^ mice: develop inflammatory lesions easier, B-cell immune system defects	Induced by IL-4, IL-12, IL-17α, and TNF-α; inhibited by IFN-γ and TGF-β
2019	(18)	Mouse	Metrnl treatment in Crohn’s disease-like mouse: ameliorate mesenteric lesions *via* STAT5/PPAR-γ	Decrease inflammatory score and pro-inflammatory cytokines
2020	(19)	Mouse	Intestinal epithelial cells Metrnl-KO mouse: deteriorate ulcerative colitis *via* autophagy-related AMPK-mTOR-p70S6K pathway	Increase TNF-α, IL-6, and IL-1β
2020	(20)	*In vitro*	Metrnl overexpression in H9C2 cells: attenuate cardiomyocytes apoptosis *via* AMPK-PAK2	Inhibit inflammation and endoplasmic reticulum stress
2020	(21)	Mouse; *in vitro*	Improve muscle regeneration by infiltrating immune cells *via* Stat3/IGF-1	Induce macrophage differentiate into inflammatory phenotype
2021	(22)	Mouse	Metrnl treatment in non-obese diabetic mice: delay onset, ameliorate islet lymphocyte infiltration	Increase IL-4, IL-10, and Foxp3; decrease IL-2, IL-17, and IFN-γ; suppress Treg cells
2021	(23)	Mouse; *in vitro*	–	Inhibit IL-1β in macrophages; associated with NLRP3 inflammasome activation
2021	(24)	*In vitro*	LPS-treated cells: ameliorate endothelial inflammation *via* AMPK and PPARδ- pathways	Suppress TNF-α, MCP-1, NFκB and IκB phos-phorylations, and inflammatory markers
2022	(25)	Mouse; *in vitro*	Impair the maturation and functions of DCs, block the development of airway hyper-reactivity	Decrease DC-mediated Th2 immune responses and inflammation
2022	(26)	*In vitro* (fish)	Up-regulate Type 1 immune response	Increase IL-1β, IL-6, IL-8, IL-17A and TNF-α

**Table 2:** Metrnl-related circulation levels and tissue expressions of human clinical studies on inflammation and immunology.

Year	Ref.	Methods	Diseases	Main findings
2019	(27)	Tissue expression	PsA, RA, OA	Highly expressed in PsA synovial tissue and fluid than OA and RA
2020	(28)	Tissue expression	Malignant mesothelioma	Immunoreactivity more prominent than mesothelial hyperplasia
2020	(29)	Serum level	Inflammatory bowel disease	Lower in UC and CD patients than controls
2020	(30)	Tissue expression	NAFLD	Hepatic Metrnl expression decrease after bariatric surgery
2021	(31)	Serum and synovial fluid levels	OA	Lower serum levels and higher synovial fluid levels in OA patients than non-OA controls
2022	(32)	Tissue expression	Invasive ductal breast cancer	Immunoreactivity increased than normal breast tissues
2022	(33)	Tissue expression	Basal cell carcinoma	Immunoreactivity increased than control and trichoblastoma
2022	(34)	Tissue expression	Serous ovarian tumors	Immunoreactivity deteced in parenchymal areas of cancer tissues, localized in epithelial areas of normal tissues
2022	(35)	Serum level	Graves’ disease	Lower in GD patients than healthy control
2022	(36)	Serum level	RA	Higher in RA patients than controls
2022	(37)	Gene expression	Bariatric surgery, obese	Increased significantly after bariatric surgery

### Metrnl and immune system

Metrnl was first described in the immune field when Albert et al. (9) sought to discover a novel or uncharacterized gene associated with the immune system through bioinformatics analyses of the BIGE database (a comprehensive human gene expression database) (38, 39). Therefore, Metrnl was identified, and it is strongly produced by various macrophages. Importantly, Metrnl^−/−^ mice exhibited B-cell immune system defects, including lower serum IgG levels (mainly because of IgG2b and IgG3 low levels) (17). Besides, its splenocytes showed an inability to secret some chemokines like CCL3 and CCL4, as well as a significant reduction in IFN levels. This study also reported that Metrnl could inhibit the expression of class MHC-II in peritoneal macrophages during IFN-γ induction (17). According to the ImmGen Datasets (https://www.immgen.org/), Metrnl could be produced by thymic medullary epithelial cells, suggesting Metrnl may also affect T-cell development.

While exploring specific mechanisms, Metrnl seems to be associated with Type 2 immune responses. Rao et al. (15) observed that Metrnl overexpression could trigger Type 2 immune cascade activation and secret cytokines IL-4/IL-13, promoting M2 macrophage activation to produce catecholamines (40). In an allergic asthma mice study, Metrnl could impair dendritic cells (DCs) maturation and function of antigen presentation both *in vitro* and vivo, thereby reducing Type 2 inflammatory responses (25). This study provided a novel treatment strategy for targeting Metrnl in allergic asthma. Besides, Legaki et al. (41) have recently identified Metrnl as one differentially methylated loci biomarker in asthma or rhinitis, which provides epigenetics evidence of Metrnl’s involvement in asthma molecularly mechanisms. It is noteworthy that injecting recombinant Metrnl could not affect B-cell class switching or production of IgE but significantly decreased T-cell proliferation if coculturing splenic CD4^+^ T cells with bone marrow-derived DCs (25). Interestingly, human helminth infections shared similar pathways with allergic asthma, a change in Type 2 phenotype differentiation (42, 43). In this state, Th2 cells develop a PD-1/PD-L2-dependent intrinsically hypo-responsive phenotype, denoted by parasite killing or impaired functionality. Furthermore, microarray data analysis revealed that Metrnl was identified as one immune regulatory gene associated with Th2 cell-intrinsic hypo-responsiveness transcriptional changes (44).

Given the immunomodulatory properties of Metrnl, it may influence tumorigenesis. Actually, Metrnl exerting pro-tumor and pro-apoptotic effects has been observed in pancreatic cancer (45, 46). But studies on Metrnl and tumor immune mechanisms are fewer, while other studies only preliminarily investigated Metrnl immunoreactivity and its relation with tumor prognosis. Metrnl immunoreactivity was increased significantly in invasive ductal breast cancer tissue compared with normal breast tissues, but no difference among the breast cancer grades (32). Similar elevation was also observed in tumor and stromal tissues of basal cell carcinoma and trichoblastoma (33), malignant mesothelioma (28), and serous ovarian tumors (34). Besides, a recent study on bladder cancer identified Metrnl as a target gene in epigenetic synergistic interactions between miRNA and DNA methylation, which was associated with the survival of potential prognostic markers in bladder cancer (47). However, this study did not provide any clinical data supporting it. Interestingly, tumor growth and metastasis depend on angiogenesis, while Metrnl has been identified as a driver of heart postinfarction angiogenesis (48). It would be a promising research orientation to explore Metrnl’s roles in tumor growth, angiogenesis, invasion, and metastasis, thus determining whether Metrnl might be a target in tumor therapy.

### Metrnl and autoimmune diseases

Metrnl was involved in multiple autoimmune disorders. As Type 1 diabetes (T1DM) is occurred by autoimmune progressive attacking pancreatic beta cells, a recent study by Zhina et al. (22) observed that intravenous administration of Metrnl could postpone the onset of diabetes in non-obese diabetic mice. This study further explored the mechanism and showed that Metrnl treatment could decrease islet lymphocyte infiltration and modulate immune cell responses. It would be prospective to combine Metrnl-related research results in T1DM patients to determine the possibility as a drug agent. Recently, Gong et al. (35) detected Metrnl levels in thyroid autoimmune diseases and found that Metrnl was significantly lower in Graves’ disease (GD) patients than in healthy controls, positively correlated with CRP and WBC. Besides, Ushach et al. (9) calculated that Metrnl expression was up-regulated in rheumatoid arthritis (RA) synovial membranes by using a gene expression database of RA they constructed before (49). Further investigation proved this finding as Metrnl was significantly increased in the synovial fluid of psoriatic arthritis (PsA) and RA compared to osteoarthritis (OA) patients, and synovial biopsies also mirrored this expression (27, 50). In contrast, Metrnl levels reduced in OA serum (31). Importantly, our academic team recently reported that Metrnl serum level was higher in RA patients than in OA and healthy controls, positively correlated with disease activity indices (36). These results suggested the close relationship between Metrnl and autoimmune-associated arthritis, but the specific mechanism mediating this role remains to be found.

Using tissue microarray, Li et al. (16) found Metrnl highly expressed in the gastrointestinal tract and the intestinal epithelium. Interestingly, a study by Gholamrezayi et al. (29) reported that Metrnl circulation concentration was lower in both Crohn’s disease (CD) and Ulcerative Colitis (UC) patients and inversely related with TNF-α and IL-6. To further figure out the mechanism, scholars generate the intestinal epithelial cell-specific Metrnl-KO mice, where Metrnl levels in the gut fluid and antimicrobial peptides release decreased significantly but did not influence colon length, intestinal permeability, or mucus content. However, UC was more prone to be induced in intestinal Metrnl^−/−^ mice than in WT mice (19). In this study, intestinal Metrnl deficiency was related to reduced autophagy in epithelial cells *via* the AMPK-mTOR-p70S6K pathway. Similar to this study, Zuo et al. (18) showed that Metrnl was increased in CD mesenteric adipose tissue and that injecting Metrnl into IL-10^−/−^ mice (CD-like model) could decrease the disease activity index and inflammatory score, indicating Metrnl mediated protective effects. In this study, Metrnl activated STAT5/PPAR-γ signaling and promoted adipocyte function, thereby ameliorating chronic colitis. Collectively, maybe different tissues produce Metrnl with different environmental regulatory mechanisms, and Metrnl may be a therapeutic target for UC and CD.

## Metrnl and inflammation

**Figure 1:**
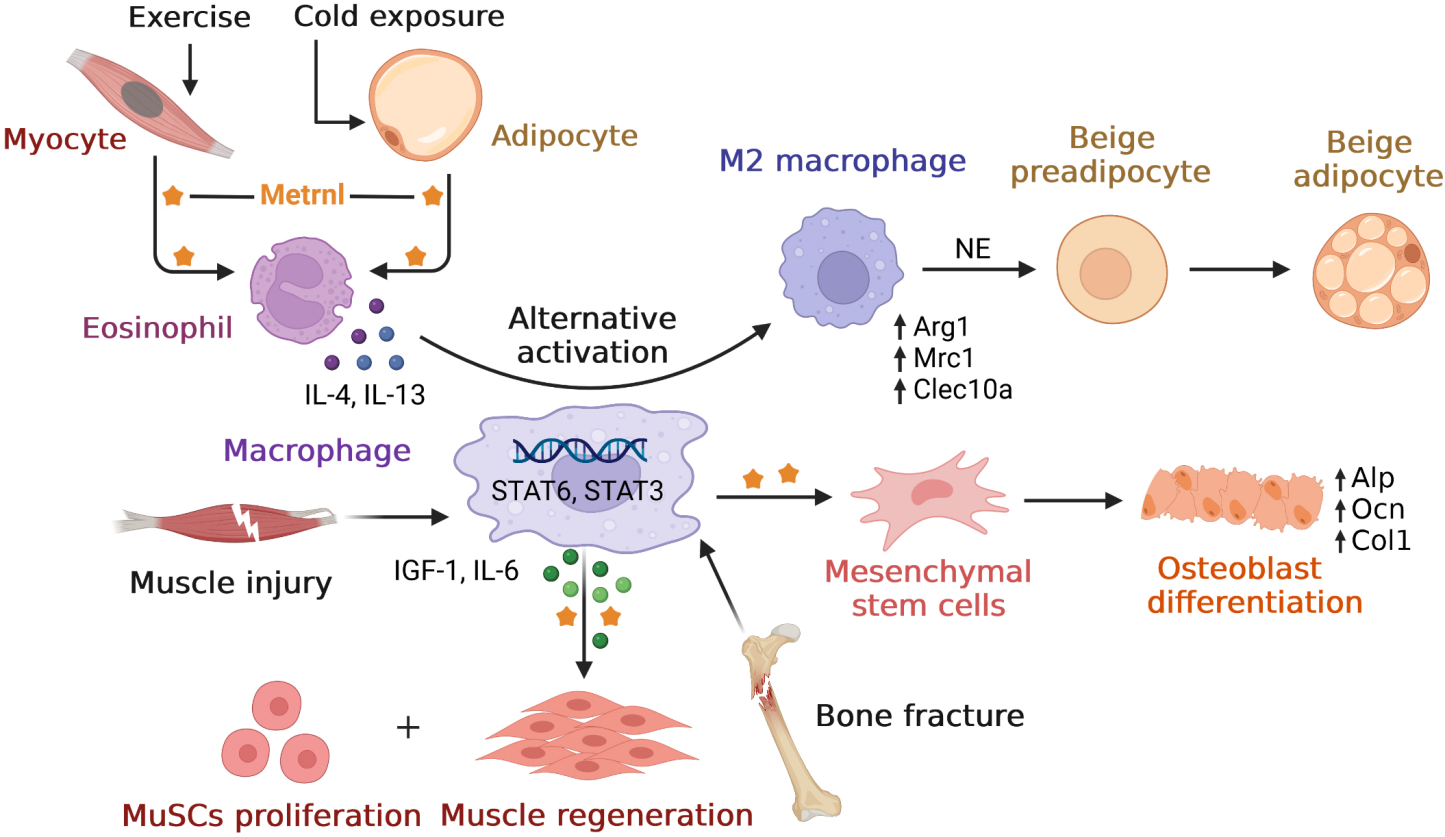
Overview of relationships between Metrnl and macrophages. Metrnl could induce IL-4 and IL-13 secretions by eosinophil upon exercise in myocytes and cold exposure in adipocytes. These cytokines stimulated macrophages’ alternative activation to M2 type *via* STAT6 signaling, which released norepinephrine (NE) and promoted adipocyte browning to increase thermogenesis. After muscle injury, macrophage-derived Metrnl activated STAT3 signal to induce IGF-1 and IL-6 secretions, thus promoting muscle stem cells (MuSCs) proliferation and skeletal muscle regeneration. Metrnl was highly expressed at regions of osteoblast active and bone depositions during bone fracture healing. Besides, Metrnl could promote mesenchymal stem cells differentiating into osteoblasts with increasing osteogenic transcripts.

Similar to inflammary factor, Metrnl also play roles in immune inflammatory response. In this part, we first overviewed Metrnl developments with inflammatory factor network in multiple diseases. Then we discussed its role of inflammation regulation in different tissues. We also summarized main findings of preclinical studies and clinical studies in inflammation (**Tables 1**, **2**). Finally, considering that Metrnl regulated several functions with the help of macrophages, we drew relationships among these relevant cells to better understand (**Figure 1**).

### Metrnl and inflammatory factor network

Many studies presented so far indicate that Metrnl is associated with inflammation. Metrnl^−/−^ mice, generated by Ushach et al. (17), were prone to develop inflammatory lesions (including uterus unilateral horn, kidneys, and liver) and endotoxin shock, suggesting Metrnl deficiency promotes inflammation development. It may play an anti-inflammatory role during sepsis. Metrnl was diffusely in activated macrophages (1, 9), as well as its production can be induced by several cytokines and inversely regulate other chemokines and cytokine production. Bone marrow macrophages can produce Metrnl by IL-4, IL-12, IL-17α, and TNF-α stimulating, of which TNF-α is the most potent inducer and can inhibit by IFN-γ and TGF-β (17). Incubating macrophages with Metrnl can also increase IL-6, IL-10, CXCL1, and CCL2 expression. Another study with similar findings by Junya et al. (26) reported that Metrnl homologues upregulated pro-inflammatory cytokines (TNF-α, IL-17A, IL-1β, IL-6, and IL-8) and promoted Type 1 immune response (IFN-γ and IL-2) *via* NF-κB dependent pathway in grass carp head kidney leukocytes. In addition, Metrnl overexpression inhibited inflammation and alleviated endoplasmic reticulum stress in injured H9C2 cells by activating AMPK-PAK2 signaling, playing an anti-inflammation role (20).

Several studies have reported that Metrnl serum levels were negatively associated with inflammatory cytokines like TNF-α and IL-6 in many metabolic diseases (20, 29, 51–53). A study on human umbilical vein endothelial cells (HUVECs) with Metrnl deficiency reported that pro-inflammatory cytokines IL-6, IL-1β, and MCP-1 were increased under normal conditions, and IL-6, IL-1β, TNF-α were elevated under ox-LDL stimulation (24). In this study, Metrnl could ameliorate LPS-induced endothelial cell inflammation through AMPK and PPAR-γ dependent pathways as endothelial injury inducing many metabolic diseases. Besides, disease activity index CRP and hs-CRP were also negatively correlated with Metrnl circulation in polycystic ovarian syndrome (PCOS), CAD, impaired glucose tolerance (IGT), GD, and type 2 diabetes Mellitus (T2DM) (35, 52, 54, 55). In contrast to these findings, two studies found that Metrnl serum levels were positively correlated with inflammation factors in chronic obstructive pulmonary diseases (COPD) and neurological disease patients (12, 56). Thus, larger participants should be involved in determining the correlation between Metrnl and the network of inflammation factors.

### Metrnl and adipose tissue inflammation

Adipose tissue eosinophils (ATEs) distribution and function are important in controlling age-related and obesity-related inflammation (57). Metrnl could stimulate an eosinophil-dependent increase in IL-4/IL-13 secreting, which promoted alternate activation of M2 macrophages to act as an anti-inflammatory function (15). Meanwhile, overexpression in Metrnl mice increases eosinophils in adipose tissue, and injecting an antibody against Metrnl prevents eosinophil accumulation (15, 40). Because Metrnl could not directly activate macrophages or adipocytes, eosinophils are indeed required for this mechanism (15). Interestingly, it is reported that transferring young mice eosinophils into an aged host can inhibit adipose tissue age-related inflammation and improve immune fitness, partially mediated by eosinophil-derived IL-4 (57). Many studies proved Metrnl can attenuate inflammation in adipose tissue (17, 18, 23, 24, 58, 59), and adipose tissue function was crucial in contributing to age-related metabolic diseases (60). Importantly, Ushach et al. (17) observed that older Metrnl-KO mice were prone to develop inflammatory lesions, especially unilateral inflammatory lesions of one uterus horn, while white adipose tissue developed no lesions. Furthermore, Metrnl played essential roles in body inflammation regulation and adipose tissue inflammation homeostasis. Beyond that, Metrnl may also slow adipose tissue inflammation associated with aging by affecting eosinophils.

### Metrnl and skeletal muscle inflammation

Increasing evidence suggests that skeletal muscle inflammation is accompanied by obesity and metabolic disorders development, which is manifested by proinflammatory activation and immune cell infiltration in intramyocellular and perimuscular adipose tissue (61). Exercise has many beneficial effects on metabolic disorders partly attributed to its anti-inflammatory effect. Metrnl, one of the myokines, can be induced in the skeletal muscle upon exercise (15), especially during muscle contractions (62). Javaid et al. (23) showed that exercise could significantly enhance Metrnl expression in various muscle depots, inhibiting NLRP3 inflammasome activation and downregulating IL-1β and IL-18 in adipose tissue. Further *in vitro* macrophage experiments also confirmed this anti-inflammation role. Moreover, Jung et al. (58) found that treating high-fat-diet (HFD) mice with Metrnl can suppress inflammatory markers and attenuate the impaired insulin response in mouse skeletal muscle and differentiated C2C12 cells.

Of note, Metrnl also played a role in regenerating damaged muscle, as it was increased sharply (30-fold) after muscle injury 24h in murine models of muscle regeneration (21). Subsequently, single-cell RNA-seq demonstrated that the most robust Metrnl transcript was by macrophage clusters in the injured muscle. Meanwhile, the LysM Cre mouse and macrophage-specific Metrnl-KO mice experiments confirmed the essential role of macrophage-secreted Metrnl in successful muscle regeneration. Moreover, Metrnl can directly signal to macrophages through Stat3 to result in differentiation to an anti-inflammatory phenotype and indirectly to primary muscle satellite cells by IGF-1 and IL-6 secretion to promote muscle stem cell expansion (21, 63). Besides, we summarized main signal pathways of Metrnl in myocytes. Collectively, Metrnl has exerted a healing and anti-inflammation effect on skeletal muscle damage, indicating that Metrnl may be a novel therapeutic target for treating aging or other inflammatory myopathies.

## Metrnl regulations in glucose metabolism

In humans, secretory proteins such as adipokines could modulate glucose uptake and release to regulate glucose homeostasis directly. Once a long-term lack of physical activities or a chronic high-fat diet induces obesity may result in disturbances of glucose metabolism, which leads to the development of insulin resistance and diabetes. In this section, we described the roles of Metrnl in glucose homeostasis, including glucose uptake, thermogenesis, and adipose browning. We also summarized regulations of Metrnl in related metabolic diseases, including insulin sensitivity, polycystic ovarian syndrome, and diabetes. Regarding controversial data in clinical studies, we analyzed it from multiple angles and summarized it in **Supplementary Table 1**.

### Metrnl and glucose homeostasis

The human body can adapt to external and internal environment variations by adjusting metabolism in peripheral tissues, such as cold exposure could induce skeletal muscle and adipose tissue a switch of glucose and fat metabolism (64). This adaptation to acute environment change might be a protective mechanism to ensure enough glucose priority for the central nervous system, whereas fatty acids and amino acids are allocated to other peripheral metabolic organs (65). Metrnl can be strongly induced in the adipose tissue upon acute cold exposure and muscle upon exercise, leading to increased energy expenditure by stimulating the browning of white adipose tissue. Unlike most adipokines identified in obesity models, Metrnl was first described in the PGC-1α4 transgenics mice model (15). PGC-1α4 is an exercise-induced splice isoform of PPARγ coactivator 1a, and skeletal muscle-specific PGC-1α4-KO transgenics mice were lean and showed an increase in basal energy expenditure (66). Given that adipose thermogenesis can augment whole-body energy expenditure, this study analyzed adipose tissues for expression of genes related to thermogenesis or involved in imparting adipose tissue browning, so METRNL was the candidate gene (15). Besides, Metrnl may promote thermogenesis function through activations of the UCP family in brown adipose tissue (67). Recently, Şekerci et al. (68) reported that central administrating of Metrnl could activate hypothalamus-pituitary-thyroid axis hormones *via* peripheral UCPs. UCPs and PGC-1α were typical thermogenic genes in the nucleus, so Metrnl played roles in body energy homeostasis regulation.

Metrnl’s ability to stimulate the browning of white adipose tissue seemed not to act directly on adipocytes but was dependent on the eosinophils-mediated IL-4/IL-13 signaling cascade of M2 alternately activated macrophages (15). This academic team subsequently demonstrated that eosinophils are indeed required for Metrnl-induced brownin. Owing to this mechanism, Metrnl has been distinguished from other adipokines with similar functions, such as irisin (69), asprosin (70), and Fgf-21 (71), as they directly stimulated adipocytes to thermogenesis. Furthermore, Zhiyong et al. (59) used inguinal subcutaneous white adipose tissue in adipocyte-specific Metrnl-KO mice to verify these effects. However, there existed no Metrnl-induced adipose browning or no thermogenesis-associated gene change. Notably, the former report showed that Metrnl-induced adipose browning occurred briefly and disappeared in one week (15). So maybe using different experimental models (relatively acute and chronic models) caused this contradiction.

Both modest cold exposure and exercise could promote the conversion of white to brown adipose tissue, increasing the body’s metabolism and helping weight loss (72). Metrnl could adapt to cold temperatures by regulating immune-adipose interactions to increase thermogenesis. Interestingly, a recent study based on Zebrafish using CRISPR/Cas9 system knockout gluk2, a protein that perceived cold in the periphery sensory neuron, revealed Metrnl was one of the differentially expressed genes (73). Although cold exposure induces no-shivering thermogenesis, long-term periods of negative caloric balance would diminish physiological resilience. In a recent study focusing on athletes in extreme cold during continuous physical activity, Coker et al. (74) observed that Metrnl serum levels remained stable throughout the 430-mile distance in temperature -45°C. In contrast to this finding, Saghebjoo et al. (75) investigated serum Metrnl changes in exercises with different temperature conditions on overweight young women. They observed that Metrnl was increased when exercising in temperate and warm water and decreased in cold water. Taking effects that the duration of exposure temperatures, the intensity of exercise, and the physical fitness of participants also affect the response of Metrnl, further investigations are required to clear appropriate temperature to enhance Metrnl expression.

### Metrnl and insulin sensitivity

Insulin resistance is a risk factor for many metabolic disorders such as obesity and diabetes. Adipose tissue regulates insulin function *via* secreting adipokines and sequestering lipids, which or else would accumulate in other tissues and produce bad effects (76). Actually, the major participant to insulin resistance is exact the excessive deposition of lipids in other organs (77). Therefore, adipokines play crucial roles in lipid-associated insulin resistance. Researchers have established many animal models to explore the relationship between Metrnl and insulin resistance. In specific-adipocyte Metrnl-KO mice, though the plasma concentration of Metrnl remained unchanged, Metrnl expression was increased in adipose tissue with its phenotypes of insulin resistance induced by high-fat-diet exacerbated a lot (59). On the contrary, overexpression Metrnl of adipocyte-specific mice could antagonize insulin resistance induced by HFD or hyperphagia (leptin knockout). These results supported that Metrnl was associated with mice’s overall insulin resistance.

**Figure 2:**
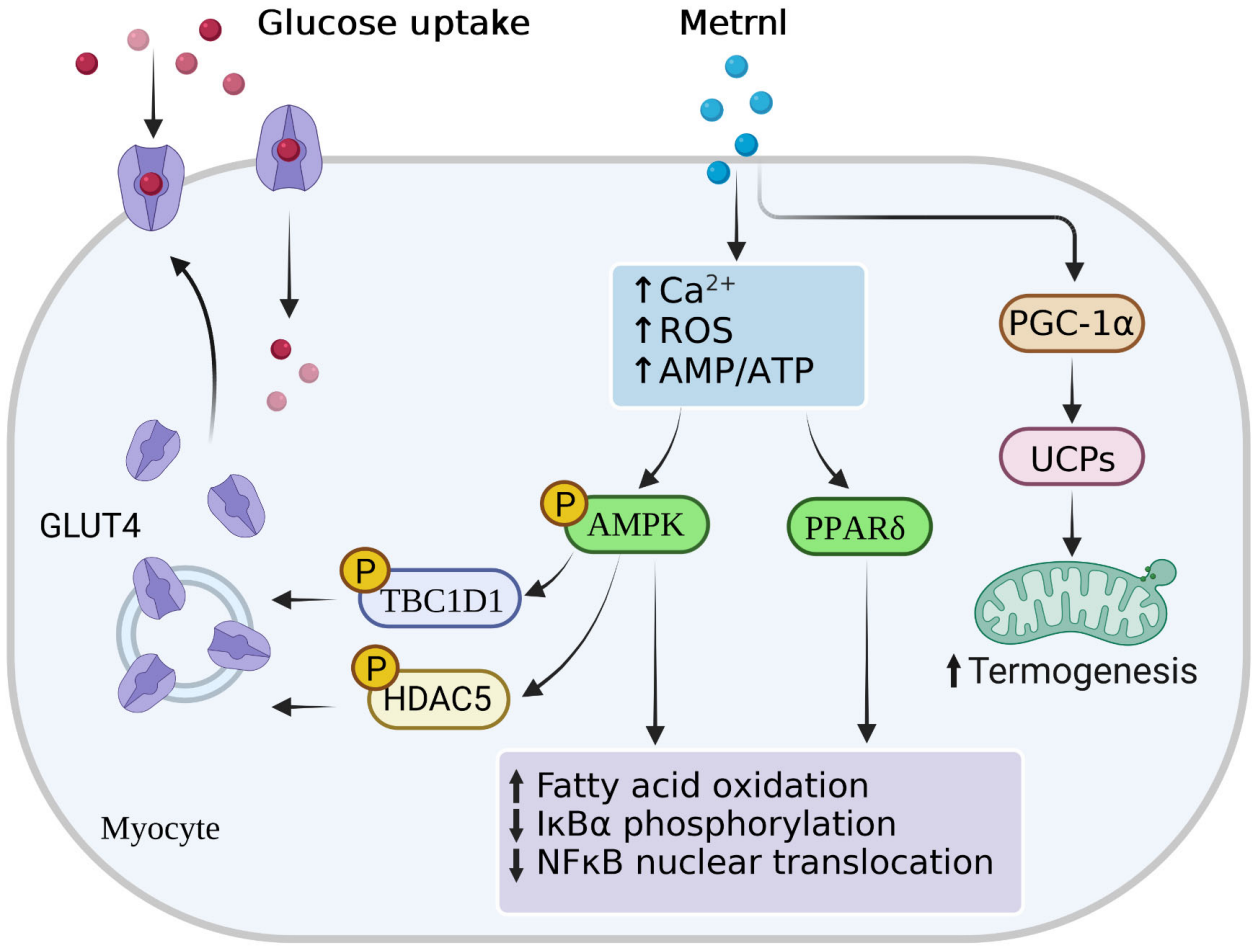
Main signal pathways of Metrnl in myocytes. Metrnl could activate AMP-activated protein kinase (AMPK) and peroxisome proliferator-activated receptor-δ (PPAR-γ) signaling by increasing intracellular calcium ion, reactive oxygen species (ROS), or AMP/ATP ratio levels in skeletal muscle cells. Activation of AMPK phosphorylation stimulated phosphorylation of HDAC5 and TBC1DI, both of which resulted in the GLUT4 transcription activation and translocation from the cytoplasm to the membrane. Highly PPAR-γ expressions and AMPK phosphorylation increased fatty acid oxidation, IκBα phosphorylation, and NFκB nuclear translocation. Besides, Metrnl also increased intramuscular PGC-1α and UCPs expressions, thus promoting mitochondria thermogenesis.

Recently, in an injecting recombinant Metrnl to treat HFD-fed mice study, Metrnl ameliorated the impaired insulin response in skeletal muscle and C2C12 myoblast cells *via* AMPK/PPARδ-mediated signaling (58). Similar studies also concentrated on T2D mice; injection of Metrnl protein can reduce the high glucose-induced insulin secretion and promote the islet β-cell function recovery by activating pancreatic islet β-cell proliferation in mice and inhibiting β-cell apoptosis (78). This function was achieved *via* the WNT/β-catenin pathway, where β-catenin could protect apoptosis of islet microvascular endothelium role (79), which also pointed to the importance of the WNT/β-catenin pathway in maintaining β-cell function. In addition, Lee et al. (62) found that Metrnl treatment with C2C12 myoblasts could increase glucose uptake *via* the calcium-dependent AMPKα2 and p38 MAPK pathways, as well as in an AMPKα2-dependent manner to regulate the binding of HDAC5 to the GLUT4 promoter. In this study, Metrnl treatment could not elevate glucose tolerance in AMPK β1β2-KO mice, demonstrating the necessity of the AMPK pathway. Notably, Wang et al. (80) reported that serum Metrnl level was correlated with insulin resistance merely, but not with β-cell function (**Figure 2**). These animal studies suggested that Metrnl has strong insulin sensitization and antagonizes insulin resistance *in vitro*; its specific dosage, duration, effects, and mechanism need further study.

HOMA-IR (Homeostatic Model Assessment of Insulin Resistance) can quantitatively assess how much insulin your pancreas needs to keep your blood sugar levels in check (81). Although HOMA-IR is an indirect measure by calculating body fasting glucose and fasting insulin levels, it is still the most popular used model in clinical research as a direct measure is impractical (82). Many clinical researchers have analyzed the correlation between Metrnl and insulin resistance in diabetes or prediabetes *via* calculating this index, but the results seemed a little controversial. A study on healthy Iranian adults found plasma Metrnl in recreational athletes increased significantly compared to sedentary subjects after correcting the insulin resistance degree (83). In contrast, baseline plasma Metrnl remained no different. Corresponding to the former study, another report on T2DM patients observed that after adjusting for HOMA-IR, Metrnl did not correlate with increased OR for T2DM. Besides, this study concluded an increase in Metrnl levels in T2DM compared to the control (84). However, most recent clinical studies reported that serum Metrnl demonstrated a negative correlation with fasting insulin and HOMA-IR, as well as T2DM patients with lower Metrnl levels (51, 85–87).

### Metrnl and polycystic ovarian syndrome

Polycystic ovarian syndrome (PCOS) is a women’s endocrine condition with more susceptibility to insulin resistance and T2DM, of which one common treatment is insulin sensitizers (88). Considering Metrnl may play roles in insulin resistance and insulin resistance is a common finding in PCOS, two independent research teams analyzed the plasma level of Metrnl in PCOS. They both report that Metrnl level was decreased in PCOS and showed a negative correlation with insulin resistance, in contrast to changes in insulin resistance markers and FSH (54, 89, 90). Moreover, in studies of patients with gestational diabetes mellitus in women of a similarly child-bearing period, Metrnl levels were significantly elevated between 24 and 28 weeks of pregnancy than normal gestation (91). Similar results were also confirmed in maternal and cord blood samples in the later stage of pregnancy; nevertheless, these increased Metrnl effects disappeared sharply after delivery (92). Taken together, a deeper understanding of the glucoregulatory mechanism of Metrnl may reveal novel strategies to treat insulin resistance and other metabolic disorders.

### Metrnl and diabetes

In line with Metrnl ameliorating insulin resistance both at animal and cellular levels, many clinical studies have also reported correlations between Metrnl circulation levels and diabetes participants with contradictory results (**Supplementary Table 1**) and regarded Metrnl as an independent risk factor of T2DM. In newly diagnosed T2DM individuals, several studies reported that serum Metrnl showed lower levels than normal glucose tolerance (NGT). Besides, Metrnl was negatively correlated with a glycemic index (including HbA1c, HOMA-IR, FBG, fasting C-peptide) and positively correlated with HOMA-β (52, 85, 93–98). Another study reported this negative correlation in long-standing diagnosed T2DM patients (85). While after 12 weeks of metformin treatment, though Metrnl remained no changes, HbA1c and FBG reduced a lot (99). Interestingly, statins medicine therapy positively correlated with Metrnl level (94). It seems the duration of diabetes has little effect on Metrnl; thus, Metrnl may not directly reflect blood glucose fluctuations in T2DM. Besides, different drug therapy may influence Metrnl secretion.

However, Wang et al. (84) found Metrnl was elevated in prediabetes and was highest in T2DM compared to IGT and NGT. Similar findings were also reported by Chung et al. (100), Wang et al. (80), and Cherian et al. (101). Notably, part of these reports lacked indicator data concerning insulin resistance and insulin sensitivity, which was challenging to explain the negative correlation between blood Metrnl and FBG. Furthermore, a study on healthy Iranian adults also concerned with FBG showed a negative correlation with baseline plasma Metrnl (83). It is worth noting that Metrnl was found to be lower in COPD patients with comorbidities such as T2DM and CAD, instead, higher Metrnl level in protopathic exacerbations (56). Dadmanesh et al. (51) noticed conflicting data regarding Metrnl circulating levels in T2DM and put to serious evaluations concluding results that Metrnl was lower and showed an independent association with the risk of T2DM presence. More recently, Wu et al. (102) used a meta-analysis indicating no significant difference between T2DM and NGT individuals but found circulation Metrnl level was lower in subgroups with HOMA-IR ≥ four and age ≤ 50 years, while higher in subgroups with BMI < 25 kg/m^2^. Collectively, this result may interfere with glucose-lowering drugs, duration of disease, sample size, age, BMI, and HOMA-IR; therefore, further longitudinal studies are required to clarify the relationship.

As diabetic nephropathy (DN) with impaired kidney function is the leading cause of end-stage renal disease, a study by Wang et al. (103) compared serum Metrnl levels in healthy controls and T2DM patients with normoalbuminuria, microalbuminuria, or macroalbuminuria, showing that all T2DM subgroups were significantly decreased, with even lower levels in macroalbuminuria group. This study reported negative associations between circulating Metrnl and BUN, Cr, GFR, ACR, CCB treatment, and ACEI/ARB treatment. Similar negative correlations with Metrnl and indicators of renal function were also reported in diabetes by Chung et al. (100). These data suggested that circulation Metrnl levels may be involved in the progression of renal disease in T2DM; however, further studies are required to figure out such potential associations.

## Metrnl regulations in lipid metabolism

Regulating the distribution of lipoprotein particles is crucial in maintaining body lipid metabolism, manifested by the liver producing and clearing lipids. Imbalances of lipoprotein would accelerate the accumulation of cholesterol in blood peripheral tissues, which leads to cardiometabolic disorders. In this part, we first concluded the direct effects of Metrnl on adipose tissue and adipocytes. Then we described the roles of Metrnl in lipoprotein homeostasis and lipid-associated diseases (**Table 3**). Finally, we introduced new findings of Metrnl therapeutical effect on cardiac diseases.

**Table 3:** Key findings of preclinical studies on the metabolism of Metrnl.

Year	Ref.	Animal/Cell models	Key findings
			**Glucose**
2014	(15)	Normal	Promote white fat browning, increase thermogenic in adipose tissue, improve glucose tolerance
2015	(59)	HFD-induced obese	Antagonize insulin sensitivity at least *via* PPARγ pathway
2018	(58)	HFD-induced obese	Improve impaired insulin functions, rescue glucose intolerance, reduce weight gains
2020	(62)	Obese, diabetes	Improve glucose metabolism *via* calcium-dependent AMPKα2/GLUT4/HDAC5
2021	(22)	Non-obese diabetes	Delay the onset of diabetes, improve islet function
2021	(78)	Diabetes	Ameliorate pancreas islet β-cell functions *via* WNT/β-catenin pathway.
			**Lipid**
2017	(104)	Human adipocytes	Promote adipose tissue accumulation, inhibite adipocyte differentiation, decrease during adipogenesis
2018	(105)	HFD-induced obese	Decrease fat accumulation
2020	(20)	H9C2 cells	Decrease myocardial ischemia/reperfusion injury and endoplasmic stress *via* AMPK-PAK2
2020	(106)	Knockout mice	Adipose Metrnl regulate blood TG, liver Metrnl regulate HDL-C
2020	(107)	Cardiac injury	Attenuate oxidative damage, apoptosis, and cardiac dysfunction *via* cAMP/PKA/SIRT1 pathway
2021	(95)	3T3-L1 cells	A transient reduce during adipocyte differentiation, decreased by omega-3 and omega-6 fatty acids
2021	(108)	Cardiac hypertrophy	Prevent cardiomyocyte hypertrophy and dysfunction, autocrine actions, a biomarker of heart failure
2022	(48)	Cardial infarction	Promote angiogenesis and infarct repair, a high-affinity ligand of KIT receptor
			**Bone and skeletal muscle**
2020	(21)	Muscle injury	Promote muscle regeneration, regulate muscle satellite cells proliferation
2022	(109)	Bone fracture	Osteoinductive, promote bone fracture healing, strongest expression by osteoblasts

### Direct effects on adipose tissue and adipocytes

Considering adipocytes represent the most crucial lipid-storing cell type, and Metrnl was expressed highly in subcutaneous adipose tissue (1); therefore, investigating Metrnl’s direct effects on adipose tissue and adipocytes is essential. Metrnl can regulate adipocyte differentiation (59, 95, 104), and its expression could reduce during human adipogenesis (104). In mice of 3T3-L1 adipocytes and human SGBS cells model, overexpression of Metrnl inhibited pre-adipocytes in differentiation to mature adipocytes and promoted cell proliferation. In contrast, adipocyte-specific knockout of Metrnl upregulated adipocyte mature-specific markers and promoted lipid accumulation but had no significance on proliferation (59, 104). Notably, silencing adipocyte PPARγ or using PPARγ inhibitors demonstrated a decrease in Metrnl expression. This study suggested PPARγ was one of Metrnl signal pathways regulating adipocyte phenotypes, but the specific mechanism needed further exploration.

Although Metrnl was upregulated in pre-adipocytes and mature adipocytes with a transient decrease during adipocyte differentiation, active substances, including glucose, insulin, and fatty acids, stimulated adipocyte Metrnl expression with no effects, whereas omega-3 and omega-6 fatty acids inhibit Metrnl expression (95). In mice studies, overexpression of Metrnl increased the abundance of beige adipocytes and revealed a robust increase in thermogenic and β-oxidation gene programs (15). Similarly, the administration of recombinant Metrnl to HFD-induced mice model also stimulated the expression of broad beige thermogenic gene programs with accompanying weight loss (15, 40). Combining Metrnl-induced browning with these results indicated that Metrnl could be regarded as a classical adipokine to investigate its physiological mechanism further.

### Metrnl and lipoprotein homeostasis

The distribution of lipoprotein particles is essential for maintaining human lipid homeostasis, whereas breaking this balance would lead to cholesterol imbalance and even a variety of diseases. Some clinical studies have reported that Metrnl was independently associated with adverse blood lipid parameters in metabolic diseases, including negatively correlating with triglyceride (TG), total cholesterol (TC), and low-density lipoprotein cholesterol (LDL-C) and positively correlating with high-density lipoprotein cholesterol (HDL-C) (55, 99, 100, 110, 111). In order to figure out the overall effects of Metrnl on clinical blood lipids, Qi et al. (106) generated global and tissue-specific knockout of Metrnl mice models. In this study, global-KO Metrnl didn’t alter any lipid parameters under a normal chow diet but increased blood TG by 14%, TC by 16%, and HDL-C by 24% upon a high-fat diet. Consistent with these findings, Li et al. (59) have demonstrated adipose Metrnl could participate in TG metabolism as its deficiency deteriorated HFD-induced acute hypertriglyceridemia. In contrast, its overexpression in adipose tissue improved TG tolerance. Interestingly, liver-specific knockout of Metrnl reduced HDL and TC but didn’t decrease VLDL release, similar to deficiency of total Metrnl (106). Moreover, intestine-specific knockout of Metrnl did not influence HDL or TC. These results imply that blood TG was at least partly modulated by adipose Metrnl rather than liver or intestine, as well tissue-specific Metrnl may control different blood lipid components.

### Metrnl and atherosclerosis

Lipid metabolic abnormality is a significant risk factor for atherosclerosis (AS), closely associated with endothelial cell damage and dysfunction. In AS mice models, Metrnl mRNA was significantly decreased in the aorta while still highly expressed in healthy controls (112). Some studies found circulation Metrnl levels were reduced in AS and coronary artery disease (CAD) patients and negatively correlated with endothelial parameters, including baPWV, ICAM-1, VCAM-1, and E-selectin (51, 52, 94, 113). Moreover, circulating Metrnl was lower in elderly patients with chronic heart failure (CHF) and negatively correlated with cardiovascular mortality, CHF rehospitalization, and combined major adverse cardiac events (MACEs) (113). Of note, a previous study has demonstrated that Metrnl could ameliorate LPS-induced endothelial cells’ inflammatory response (24). Consistent with these findings, based on a large-scale vascular adhesion molecules Genome-Wide Association Study (GWAS), Metrnl was identified as a candidate gene associated with carotid intima-media thickness (cIMT) in whole blood gene expression (114). cIMT is a new biomarker of subclinical AS and a predictor of impending cardiovascular events, where indicating Metrnl may also involve in AS development (115). It seemed endothelial impairment factors could facilitate Metrnl secretions by endothelial cells, and circulation Metrnl levels decreased significantly with the AS progression. Moreover, areas of aortic plaques, necrotic injuries, and lipid accumulations of aortic root were heavier in aorta plaque of endothelial Metrnl deficiency mice models than those in control, with more severe spontaneous atherosclerosis (112). These results suggested that the AS mechanism is related to Metrnl deficiency in endothelial cells.

Many studies have reported that Metrnl was a novel myokine with protective effects on cardiovascular diseases. Two previous studies have detected Metrnl abundantly expressed in cardiac muscle (15, 59), as well following Hu et al. (107) found it significantly decreased upon doxorubicin (DOX)-induced cardiotoxicity exposure. This study further found Metrnl could exert cardioprotective effects *via* activating the cAMP/PKA/SIRT1 pathway as cardiac-specific overexpression of Metrnl markedly improved mice cardiac dysfunction and survival status, while Metrnl deficiency aggravated cardiac injury. Of note, Metrnl could not affect the tumor-killing capacity of DOX (107). Rupérez et al. (108) also confirmed a similar conclusion: they constructed Metrnl-KO mice exhibiting asymmetrical cardiac hypertrophy, fibrosis, and aging. Conversely, overexpression of heart Metrnl could prevent cardiac remodeling. In addition, this study used cardiomyocytes *in vitro*, demonstrating that Metrnl could inhibit cardiac hypertrophy development, indicating a direct effect on cardiac cells.

### Metrnl and heart regenerative

Although the human heart has limited regenerative capability after damage, stimulation of new blood vessel formation is an essential part of the reparative process and might be a potential approach to ameliorate heart function. When recognizing that cell-cell communications between cardiac myeloid cells and other nonmyocytes might participate in heart injury (116), Reboll et al. (48) found that Metrnl protein may serve as a potential mediator aspect of this cellular cross-talk. Notably, previous studies have noticed Metrnl expression was prominently increased in mouse infarcts and patients’ tissue with acute myocardial infarction, but circulating Metrnl level was decreased (117). In mice with a cardiac attack, monocytes and macrophages migrated to the heart, producing Metrnl to stimulate the expansion of vascular endothelial cells, resulting in an angiogenic response that limited damage (118). Besides, delivering Metrnl protein with an infusion pump into the mouse could promote angiogenesis after acute myocardial infarction. More importantly, Metrnl was found as a ligand of KIT receptor tyrosine kinase and could respond to the secreted protein stem cell factor (SCF) (119). The activation of KIT is necessary for the normal angiogenic response after cardiac infarction, and SCF is the function of maintaining KIT-expressing hematopoietic stem cells during development, both of which could stimulate the expansion of KIT-expressing mice endothelial cells (120). Accordingly, the role of METRNL-KIT signaling would open new avenues for developing therapies for cardiac disease.

## Metrnl regulations in bone metabolism

### Metrnl and osteoblasts

Many studies have reported that Metrnl was involved in skeletal development, remodeling, and some bone-related diseases. After constructing a human osteoblast full-length cDNA library, Gong et al. (121) screened unreported genes closely related AP-1 transcription complex and identified Metrnl as the only candidate gene. In multiple parts of mouse bones, RNA-seq demonstrated the strongest expression of Metrnl transcript by osteoblast. Meanwhile, Metrnl deficiency would decrease mice’s osteogenic capacity in bone marrow stromal cells, suggesting that Metrnl is abundant in bone deposition areas where osteoblasts differentiate and activate (109). Besides, Huang et al. (109) found that Metrnl could increase osteoblast differentiation and mineralization *in vitro* and promote bone fracture healing. Notably, knockdown of the Metrnl gene did not affect overall skeletal development, fracture healing, or osteoblasts in the animals in this study. Nevertheless, it seemed overexpression of Metrnl would inhibit mineralized nodule formation (121). Recently, Cherian et al. (101) observed a strong positive association between serum Metrnl levels and various molecules with osteogenic properties in obesity and diabetes patients, suggesting Metrnl may potentially affect bone-related development complications.

Although Metrnl could affect osteoblasts both *in vitro* and vivo, considering the effects of Metrnl on inflammation, some studies regarded Metrnl play roles after skeletal injury precisely with the help of macrophages (17). Similar results were also observed in studies of skeletal muscle adjacent to skeletal anatomy, where Metrnl was highly expressed in the early stages after muscle damage while knocking out the Metrnl gene did not result in changes in various muscle properties either (21). Interestingly, many investigators noticed that numerous factors from local circulation were able to alter bone regeneration (122–124). Consequently, this academic team proposed a compensatory mechanism where Metrnl may be a compensating molecule lacking *in vitro* culture models. This would explain why Metrnl was dispensable for both bone and muscle development or healing *in vivo* but served as an osteoinductive molecule and an effector of skeletal muscle regeneration *in vitro* (63, 109). Taken together, supplementing Metrnl at cellular levels may potentially improve osteoblast function. Besides, further investigations are needed to understand specific mechanisms of Metrnl in osteogenic differentiation and how the body delivers Metrnl into sites of active bone formation.

### Metrnl and skeletal-related diseases

Some studies have reported that Metrnl was expressed abnormally in the cartilage tissue and synovium of skeletal-related diseases. In patients with OA, serum Metrnl levels were significantly lower than normal healthy controls but elevated in synovial fluid (31). This confirmed a hypothesis that Metrnl in different tissues might have different environmental mechanisms. Similarly, in two gene expression profiles of cartilage tissue, compared with healthy cartilage from patients with traumatic femoral neck fractures alone, Metrnl was strongly downregulated in OA patients (ratio = 0.34) but upregulated in non-traumatic osteonecrosis of femoral head (NOFH) (ratio = 11.77) (125, 126). These revealed the Metrnl gene involved in pathogenetic differences in NOFH and OA cartilage damage, and it would be interesting to explore the mechanism further. Besides, as we mentioned in the previous section of Metrnl and autoimmune diseases, Metrnl was elevated in synovial fluid of PsA and RA compared to OA patients, and our team also found serum Metrnl levels were higher in RA with a positively correlated with disease activity indices (27, 36, 50). Collectively, the mechanism of Metrnl in skeletal-related diseases merits further research, especially bone-related autoimmune diseases, because immune function interacts with the homeostasis of the skeletal system’s internal environment closely.

## Metrnl regulations in obesity and exercise

Obesity is a worldwide health problem with an increased prevalence of metabolic-related disorders. Exercise is an effective strategy to prevent and treat obesity and its related metabolic syndromes. It is well-documented that changes in circulating cytokines could occur after exercise but with unclear mechanisms. Here we concluded circulation Metrnl levels in obese merged with or without metabolic diseases and discussed Metrnl involvements in bariatric surgery (**Supplementary Table 1**). We also discussed the roles of Metrnl after exercise and compared circulating Metrnl changes upon different exercises.

### Metrnl and obesity

Obesity is an important promoter of chronic and low-grade inflammation states with lipid accumulation, which results in many metabolic syndromes (61, 127). Previously, Li et al. (1) discovered that Metrnl was upregulated in circulation and adipose tissue of HFD-induced obese mice. Similarly, Löffler et al. (104) reported that Metrnl was highly expressed in adipocytes of obese compared to lean children. These interactions with adipose tissue dynamics indicated the association between Metrnl and obesity. However, correlations between serum Metrnl level and obesity indexes were contradictory. Some studies reported that simply obese individuals (29, 85, 103, 128) or obese patients with metabolic syndrome have been shown lower Metrnl levels and were negatively related to body mass index (BMI) (51, 54, 55, 85, 92, 94, 103, 129). Notably, this negative association was prone to arise in T2DM patients compared with other diseases. Actually, it seems more studies figured out that there is no relationship between Metrnl and BMI in healthy or metabolic syndrome (31, 52, 80, 83, 87, 89, 95, 99, 100). Apart from total height and body weight, the waist-to-hip ratio (WHR) was further measured but still no correlation (52). Considering visceral fat obesity (VFO) is close to obesity and insulin resistance, there were few studies on the correlations between Metrnl and VFO, as most obesity evaluations focused on measurements of waist circumference or BMI. Du et al. (111) used Dual Energy X-ray Absorptiometry (DXA) to quantify the visceral fat area and found that Metrnl level was independently inversely associated with visceral fat deposition. This finding was the first and only report demonstrating the association between Metrnl and VFO, which provided a possibility that Metrnl concentration may be a useful noninvasive, cost-effective marker for assessing VFO.

Moreover, some results also found a positive correlation between Metrnl levels and BMI. An Arab survey stratified T2DM and healthy controls based on obesity and found Metrnl plasma levels elevated more in obese T2DM (BMI > 30 kg/m^2^) than in non-obese T2DM (20 kg/m^2^ ≤ BMI ≤ 30 kg/m^2^) (130). Wang et al. (84) and Cherian et al. (101) reported similar findings. This increase was probably one of the physiological regulation mechanisms to restore glucose tolerance or defense response to counteract metabolic stress. Of note, these studies have not excluded clinical confounding factors’ effects, such as diabetes-related drugs, physical activity, sex, and age. Besides, obesity or overweight could accelerate the loss of muscle mass, as Metrnl was also secreted from muscle, where different Metrnl levels might result from adipose tissue dysfunction and sarcopenia. Collectively, larger sample sizes for clinical studies and more rigorous experimental designs are needed to identify the correlation between circulating Metrnl levels and obesity, thus determining whether Metrnl could be used as a biomarker of obesity.

Bariatric surgery (BS) has been currently the most effective therapy for morbid obesity and associated complications. A recent mice model study reported that Metrnl and UCPs were changed in favor of increased thermogenesis through fat browning to induce weight loss comparing laparoscopic sleeve gastrectomy (LSG) mice group with sham surgery and pair-fed groups (131, 132). They showed a positive correlation between Metrnl and weight loss after LSG-induced weight loss in adipose tissue, muscle, and plasma. Of note, this scholarly team recently reported the same results in the human interventional study (37). A similar clinical context study was also reported by Grander et al. (30), which found Metrnl expression was decreased in hepatic-and adipose tissues after BS in non-alcoholic fatty liver disease (NAFLD). Interestingly, Metrnl has been identified as a target protein with Iah1, a candidate gene for diet-induced NAFLD (133). Maybe Metrnl as one of the hepatokines play role in NAFLD progress but further investigations are needed to clarify the mechanism. However, another study by Pellitero et al. (128) showed the opposite result, which reported that Metrnl circulation levels were increased after LSG 6 and 12 months in obese patients, whereas at baseline patients with obesity showed lower. Schmid et al. (95) also observed serum Metrnl concentrations in patients undergoing BS and a low-calorie diet were increased after 3 months and back to baseline levels after 12 months, which also exerted no gender-specific effect. Taken together, these clinical studies showed contradicting results and this difference may attribute to co-morbidities, obesity, smaller sample size, or observation time as confounding factors. Further prospective studies with better design are required to better explain the relationship between Metrnl and weight loss or obesity.

### Metrnl and exercise

Exercise-inducible soluble factors, such as adipokines, cytokines, myokines, and osteokines are regarded to play important roles in the body’s response to exercise (134, 135). Metrnl was first described by Rao et al. (15) who investigated Metrnl’s ability to moderate energy thermogenesis originally not in specific-METRNL transgenic mice but in PGC-1α4 transgenic mice. PGC-1α4 is an exercise-induced splice isoform of PPARγ coactivator 1a, where Metrnl was regarded as its downstream effector protein. A recent study reported by Amano et al. (136) supported this effector pathway of PGC-1α along with Metrnl. After they applied 4-week electrical stimulation to rats’ legs, which aimed to simulate chronic resistance exercise, an apparent positive correlation was found between the expression of PGC-1α in brown adipose tissues and plasma Metrnl levels. Similar results have been confirmed by Bae et al. (137) who exerted an 8-week training on HFD-induced obese mice increasing muscle proteins including AMPK, PGC-1α, and plasma Metrnl. Further studies proved Metrnl can be significantly induced in various muscle depots and adipose tissue by exercise (15, 23, 62). Besides, up-regulation of intramuscular Metrnl induced by regular exercise would be a pathway to suppress obesity and metabolic syndromes (105, 138, 139). Jung et al. (58) reported that administration of Metrnl in obese mice could reduce body weight gain and rescue glucose intolerance, whereas not affect calorie intake. Moreover, results from Hafiz et al. (23) found that exercise exerted anti-inflammatory function by NLRP3 inflammasome activation of HFD-induced obese mice, and this program was associated with Metrnl anti-inflammatory effects in macrophages in muscle.

A number of clinical studies have reported higher circulation Metrnl levels in response to exercise (140–146). Interestingly, exercise with different intensity, type, or duration may also influence Metrnl activity. Some studies have investigated the effects of regular exercises on obese mice, such as undergoing treadmill training, with obtaining similar conclusion that Metrnl levels increases (137). Of note, a previous study didn’t observe any changes in resting Metrnl level upon endurance training in mice (15). Given that recent research has reported beneficial effects of high-intensity interval training (HIIT) with metabolic diseases, they recently examined the impact of high-intensity interval training and short-term interval training on the Metrnl mRNA of skeletal muscle biopsy samples in 9 healthy males (147). An increase in Metrnl expression at 3-hour recovery compared to rest was reported after a single bout of high-intensity interval exercise before training, yet no statistical significance of post-training increases. It would be interesting to determine whether increased muscle Metrnl expression was a common adaptive response to certain types of exercise.

However, a number of earlier studies were based on mRNA levels, while accompanied by different commercial ELISA coming into service (93) more results were prone to support that circulation Metrnl levels can be strongly induced by both acute and chronic exercise (148). More recently, a similar study on serum Metrnl levels to investigate the different types of exercise effect (including aerobic exercise, HIIT, and resistance exercise), all exercise groups significantly elevated Metrnl levels, whereas no assessment changes within the group (149) Additionally, Amano et al. (136) figured out the chronic resistance exercise training also up-regulated Mertnl levels in obese mice. As such, findings from Alizadeh et al. (150) suggested that human downhill running exercise, regarded as a muscle-damaging exercise protocol, significantly elevated Metrnl levels as well as positively correlated with eosinophils’ number. Moreover, combined training (CT) for its metabolic benefits has been recommended by diabetes guidelines, Bonfante et al. (151) evaluated effects of 16-week-CT period on serum pro-thermogenic/anti-inflammatory inducers in overweight T2DM individuals and found Metrnl was positively correlated with brown adipose tissue (BAT) thermogenic activity. Interestingly, fed and fasting states in the pre-training or post-training also influence Metrnl secretion reported, with Metrnl was increased both in two states (152). Taken together, although some important questions remain unanswered, it would be interesting to evaluate Metrnl’s roles in the beneficial effects of exercise with metabolic diseases.

## Future perspectives and conclusions

Overall, well-documented evidence has suggested that this novel secreted protein exerts pleiotropic effects on inflammation, immunology, and metabolism. However, Metrnl is not secreted by the fixed model of cell types but depends on the concrete physiological and pathological context. Based on different tissue homeostasis, more preclinical studies are needed to clarify the molecular mechanisms that control the selective expression of Metrnl by different cell types. Meanwhile, contradictoriness in reported results highlights the necessity for more-accurate methods to measure Metrnl and for clinical studies to be better designed, revealing the role of Metrnl in different diseases. Whether Metrnl is used as a predictive biomarker or disease activity indicator for cardio-metabolic syndromes including morbid obesity, insulin resistance, T2DM, CAD, and PCOS, or other inflammatory immune diseases such as asthma, RA, OA, PsA, UC, CD, T1DM, and GD, depending on more rigorous prospective studies. Finally, based on current knowledge, trials to assess the application of Metrnl as a therapeutic agent in humans is premature but should be one of the research goals for the near future.

## Author contributions

ZL conceived this article. ZL and ZG drafted the manuscript. TS and SZ drew the illustrations and tables. SY and MZ revised the article. ZL, ZG, TS, SZ, SY, and MZ checked and edited the article. HS supervised the article. All authors have read and agreed to the final version of the manuscript.
