# Supplementary material for: Meteorin-like/Metrnl, a novel secreted protein implicated in inflammation, immunology, and metabolism: A comprehensive review of preclinical and clinical studies
Source: Front Immunol. 2023 Feb 24;14:1098570. doi: 10.3389/fimmu.2023.1098570 (PMC9998503; doi:10.3389/fimmu.2023.1098570)
Supplement: Supplementary file 1 [file Table_1.docx]

***Supplementary Material***

# Supplementary Table 1: Human clinical studies investigating the association of Metrnl with obesity, insulin resistance, diabetes, and coronary artery disease (CAD)

| **Year** | **Ref.** | **Study subjects** | **Metrnl levels associated with** | | | | **N** | **Metrnl levels also associated with** |
| --- | --- | --- | --- | --- | --- | --- | --- | --- |
|  |  |  | **Obesity (BMI)** | **Insulin resistance**  **(HOMA-IR)** | **Diabetes** | **CAD** |  |  |
| 2018 | (1) | T2DM vs Prediabetes vs NGT | **No** | **Negative** | **Negative** | NR | 139 | HbA1c, TG, TC, LDL-C, FPG, post-load 2-h glucose (negative)  HOMA-β (positive) |
| 2018 | (2) | Obesity vs Healthy | **Negative** | **Negative** | **No** | NR | 58 | OSM, HbA1c, glucose, insulin (negative) |
| 2018 | (3) | CAD vs T2DM vs Healthy | **Negative**  (T2DM)  **No** (CAD) | **Negative** | **Negative** | **Negative** | 170 | TNF-α, IL-6 (negative);  T2DM: FBG (negative);  CAD: adiponectin (positive) |
| 2018 | (4) | Healthy | NR | NR | NR | NR | 13 | exercise in different temperature water (negative) |
| 2018 | (5) | T2DM vs Healthy (Sarcopenic Obesity) | **No** | **Positive** | **Positive** | NR | 800 | DBP, SBP, TC, TG, LDL-C, BUN, Cr, eGFR, hemoglobin, FPG (negative);  T2DM: leukocyte count (positive) |
| 2019 | (6) | T2DM vs NGT | **Positive** | **No** | **Positive** | NR | 228 | HbA1c, TC (positive);  HOMA-β (no correlation) |
| 2019 | (7) | NGT vs IGT vs T2DM | **No** | **Negative** | **Negative** | NR | 260 | FPG, 2h-PLG, HbA1C, FINS, hs-CRP, IL-6, TNF-α, ICAM-1, VCAM-1, E-selectin, CIMT, baPWV (negative) |
| 2019 | (8) | NGT vs IFG vs IGT vs T2DM | **Positive** | **Positive** | **Positive** | NR | 160 | WC, TC, TG, LDL-C, FBG, PBG, HbA1c, FINS (positive);  HDL-C (negative) |
| 2019 | (9) | CAD vs Healthy | **Negative** | NR | NR | **Negative** | 349 | the severity of CAD, TC, LDL-C, hs-CRP, IL-1β (negative);  IL-11 (positive) |
| 2019 | (10) | T2DM vs NGT | **No** | **Positive** | **Positive** | NR | 89 | FBG, PBG (positive);  HOMA2-%S (negative);  HOMA-β (no) |
| 2019 | (11) | Obstructive sleep apnoea syndrome subjects vs Healthy | **Negative** | NR | NR | NR | 313 | LDL-C, apnoea–hypopnea index, number of arousals, hypopnea, apnoea (negative) |
| 2020 | (12) | T2DM | **Negative**  (VFA,VFO) | NR | NR | NR | 321 | TC, TG, LDL-C, ALB (negative);  age, height, BUN, Cr, UA(positive) |
| 2020 | (13) | T2DM vs Healthy | **Negative** | **Negative** | **Negative** | NR | 150 | FBG, HbA1c, Insulin (negative) |
| 2020 | (14) | T2DM vs Healthy | **Negative** | NR | **Negative** | NR | 295 | diabetic nephropathy, ACR, ACEI/ARB, HbA1c, BUN, UA (negative); Cr (positive) |
| 2020 | (15) | GDM vs Healthy | NR | **Positive** | **Positive**  (cord plasma) | NR | 60 | Metrnl reduced significantly at delivery in GDM; glucose, insulin, TC, TG (positive) |
| 2020 | (16) | Healthy | **No** | **Positive** | NR | NR | 40 | physical activity (positive);  glucose (negative) |
| 2020 | (17) | COPD vs Healthy | NR | NR | **Negative**  (comorbidities) | **Negative**  (comorbidities) | 313 | CRP (positive);  HbA1c (negative) |
| 2020 | (18) | T2DM vs Prediabetes vs Healthy | **Negative**  (T2DM)  **No**  (prediabetes, healthy) | **Negative** | **Negative** | NR | 138 | ICAM-1, VCAM-1, E-Selectin, HbA1c, Insulin, FPG, prediabetes (negative); statins medicine therapy (positive) |
| 2020 | (19) | IBD vs UC vs CD vs Healthy | **Negative** | NR | NR | NR | 224 | TNF-α, IL-6 (negative);  FBG (positive) |
| 2020 | (20) | PCOS vs Healthy | **No** | **Negative** (PCOS)  **No** (healthy) | NR | NR | 60 | FSH (negative);  Metrnl is low in PCOS comparing with healthy |
| 2020 | (21) | T2DM vs NGT | **Negative** | No | NR | NR | 1698 | Metrnl levels was lower in subgroups with HOMA-IR ≥ 4 and age ≤ 50 years, while higher in subgroups with BMI<25 kg/m^2^. |
| 2021 | (22) | GDM vs NGT | **No** (maternal plasma)  **Negative**  (cord plasma) | NR | **No** (maternal plasma)  **Negative**  (cord plasma) | NR | 111 | A positive correlation between fetal and maternal levels;  Average in cord blood was 2-fold higher than normal adult |
| 2021 | (23) | CHF vs Healthy | NR | NR | NR | **Positive**  (CHF) | 161 | An independent predictor of all-cause and cardiovascular death |
| 2021 | (24) | Obesity  (LCD and BS) | **No** | NR | **Negative** | NR | 312 | HDL, Leptin, Resistin (positive)；  BS: TG (negative)；  LCD: HbA1c (negative); Progranulin (positive) |
| 2021 | (25) | T2DM | **Positive** | NR | **Positive** | NR | 228 | Osteoactivin (positive);  OPG, SPARC, Syndecan-4 (no); |
| 2021 | (26) | Obesity  (LAGB) | NR | NR | NR | NR | 33 | LAGB-associated weight loss was correlated with decreased hepatic Metrnl expression |
| 2021 | (27) | OA and Obesity | **No** | **Positive** (synovial-fluid)  **No** (serum) | NR | NR | 55 | Metrnl is high in synovial-fluid comparing with serum; Metrnl is high in obese non-OA comparing with non-obese healthy;  synovial-fluid: age, TG (positive);  QUICKI (negative) |
| 2021 | (28) | PCOS vs Healthy | **Negative** (healthy)  **No** (PCOS) | **Negative** | NR | NR | 180 | Metrnl is low in PCOS comparing with healthy; hs-CRP (negative)  PCOS: FBG, fasting Insulin (negatove) Healthy: LH, Log Adiponectin (negative) |
| 2021 | (29) | IGM | NR | **No** | **No** | NR | 72 | HbA1c, glucose (no) |
| 2021 | (30) | Patients with various diseases | NR | NR | NR | NR | 260 | Serum: CRP, IgA (positive); hemoglobin, ALB (negative);  CSF: IgM, IgA, IgG, lactate, total protein, ALB, Urea, ALT, AST (positive); Cr (negative) |
| 2021 | (31) | COVID-19 vs Healthy | NR | NR | NR | NR | 86 | COVID-19: lower levels |
| 2021 | (32) | T2DM vs CAD vs Healthy | **Negative** (BMI ≥ 25) | **No** | **No** | **No** | 2577 | Meta-analysis |
| 2022 | (33) | CHF vs Healthy | **Positive** | NR | **Negative** | **Negative**  (CHF) | 1066 | LVEF (positive);  NT-proBNP, cardiovascular mortality, CHF rehospitalization, the combined MACEs (negative) |
| 2022 | (34) | MetS vs Healthy | **No** | NR | NR | NR | 90 | asprosin, visfatin (positive) |
| 2022 | (35) | T2DM vs Healthy | **No** | **Negative** | **Negative** | NR | 120 | Insulin, TG, glucose level (negative) |
| 2022 | (36) | RA vs Healthy | **No** | NR | NR | NR | 231 | RA: DAS28, RF, and CRP (positive) |
| 2022 | (37) | Healthy vs Overweight vs obesity | **Negative** | **Negative** | NR | NR | 182 | TG, TC, LDL-C, sdLDL (negative)  HDL-C (positive) |
| 2022 | (38) | STEMI vs NSTEMI vs Healthy | **No** | NR | NR | NR | 115 | NSTEMI: lower levels;  STEMI: no significance |
| 2022 | (39) | T2DM vs Healthy | **No** | **Negative** | **Negative** | NR | 120 | insulin, TG, glucose levels (negative) |

Positive, positive association; Negative, negative association; No, no association; NR, not related; T2DM, diabetes mellitus type 2; UC, ulcerative colitis; IBD, inflammatory bowel disease; CD, Crohn’s disease; LAGB, laparoscopic adjustable gastric banding; CHF, chronic heart failure; MetS, metabolic syndrome; IGM, impaired glucose metabolism; IFG, impaired fasting glucose; IGT, impaired glucose tolerance; NGT, normal glucose tolerance; GDM, gestational diabetes mellitus; OA, osteoarthritis; BMI, body mass index; WC, weight circumference; TC, total cholesterol; TG, triglycerides; LDL-C, low-density lipoprotein cholesterol; HDL-C, high-density lipoprotein cholesterol; UA, uric acid; ALB, albumin; ALT, alanine aminotransferase; AST, aspartate aminotransferase; BUN, blood urea nitrogen; Cr, creatinine; HbA1c, hemoglobin A1c; FPG, fasting plasma glucose; PBG, postprandial blood glucose; 2h-PLG, 2h postload glucose; hs-CRP, high-sensitive C-reactive protein; IL-6, interleukin-6; TNF-α, tumor necrosis factor-α; FSH, follicle-stimulating hormone; LH, luteinizing hormone; ARB, angiotensin II receptor blockers; ACEI, angiotensin-converting enzyme inhibitor; ACR, albumin to creatinine ratio; VFO, visceral fat obesity; VFA, visceral fat area; BS, bariatric surgery; LCD, low calorie diet; BP, blood pressure; SBP, systolic blood pressure; DBP, diastolic blood pressure; FINS, fasting insulin; ICAM-1, intercellular adhesion molecule-1; VCAM-1, vascular cell adhesion molecule-1; CIMT, carotid intima media thickness; baPWV, brachial-ankle pulse wave velocity; OPG, osteonectin; SPARC, steoprotegerin; QUICKI, quantitative insulin-sensitivity check index; LVEF, left ventricular eject fraction; NT-proBNP, N-terminal pro brain natriuretic peptide; MACEs, major adverse cardiac event(s); RA, rheumatoid arthritis; RF, rheumatoid factors; STEMI, ST-elevation myocardial infarction; NSTEMI, non-elevation myocardial infarction

# References

1. Lee JH, Kang YE, Kim JM, Choung S, Joung KH, Kim HJ, et al. Serum Meteorin-like protein levels decreased in patients newly diagnosed with type 2 diabetes. Diabetes Res Clin Pract. 2018;135:7-10.

2. Pellitero S, Piquer-Garcia I, Ferrer-Curriu G, Puig R, Martínez E, Moreno P, et al. Opposite changes in meteorin-like and oncostatin m levels are associated with metabolic improvements after bariatric surgery. Int J Obes (Lond). 2018;42(4):919-22.

3. Dadmanesh M, Aghajani H, Fadaei R, Ghorban K. Lower serum levels of Meteorin-like/Subfatin in patients with coronary artery disease and type 2 diabetes mellitus are negatively associated with insulin resistance and inflammatory cytokines. PLoS One. 2018;13(9):e0204180.

4. Saghebjoo M, Einaloo A, Mogharnasi M, Ahmadabadi F. The response of meteorin-like hormone and interleukin-4 in overweight women during exercise in temperate, warm and cold water. Horm Mol Biol Clin Investig. 2018;36(3).

5. Chung HS, Hwang SY, Choi JH, Lee HJ, Kim NH, Yoo HJ, et al. Implications of circulating Meteorin-like (Metrnl) level in human subjects with type 2 diabetes. Diabetes Res Clin Pract. 2018;136:100-7.

6. AlKhairi I, Cherian P, Abu-Farha M, Madhoun AA, Nizam R, Melhem M, et al. Increased Expression of Meteorin-Like Hormone in Type 2 Diabetes and Obesity and Its Association with Irisin. Cells. 2019;8(10).

7. El-Ashmawy HM, Selim FO, Hosny TAM, Almassry HN. Association of low serum Meteorin like (Metrnl) concentrations with worsening of glucose tolerance, impaired endothelial function and atherosclerosis. Diabetes Res Clin Pract. 2019;150:57-63.

8. Wang K, Li F, Wang C, Deng Y, Cao Z, Cui Y, et al. Serum Levels of Meteorin-Like (Metrnl) Are Increased in Patients with Newly Diagnosed Type 2 Diabetes Mellitus and Are Associated with Insulin Resistance. Med Sci Monit. 2019;25:2337-43.

9. Liu ZX, Ji HH, Yao MP, Wang L, Wang Y, Zhou P, et al. Serum Metrnl is associated with the presence and severity of coronary artery disease. J Cell Mol Med. 2019;23(1):271-80.

10. Wang C, Pan Y, Song J, Sun Y, Li H, Chen L, et al. Serum Metrnl Level is Correlated with Insulin Resistance, But Not with β-Cell Function in Type 2 Diabetics. Med Sci Monit. 2019;25:8968-74.

11. Sun H, Zhang Y, Wang J, Kong J. Correlation of serum meteorin-like concentration with the presence and severity of obstructive sleep apnoea syndrome. Ann Clin Biochem. 2019;56(5):593-7.

12. Du Y, Ye X, Lu A, Zhao D, Liu J, Cheng J, et al. Inverse relationship between serum Metrnl levels and visceral fat obesity (VFO) in patients with type 2 diabetes. Diabetes Res Clin Pract. 2020;161:108068.

13. Onalan E, Cavlı C, Dogan Y, Onalan E, Gozel N, Buran I, et al. Low serum levels of meteorin-like/subfatin: an indicator of diabetes mellitus and insulin resistance? Endokrynol Pol. 2020;71(5):397-403.

14. Wang R, Hu D, Zhao X, Hu W. Correlation of serum meteorin-like concentrations with diabetic nephropathy. Diabetes Res Clin Pract. 2020;169:108443.

15. Yavuzkir S, Ugur K, Deniz R, Ustebay DU, Mirzaoglu M, Yardim M, et al. Maternal and umbilical cord blood subfatin and spexin levels in patients with gestational diabetes mellitus. Peptides. 2020;126:170277.

16. Alizadeh H, Alizadeh A. Association of Meteorin-Like Hormone with insulin resistance and body composition in healthy Iranian adults. Diabetes Metab Syndr. 2020;14(5):881-5.

17. Kerget B, Afşin DE, Kerget F, Aşkın S, Akgün M. Is Metrnl an Adipokine İnvolved in the Anti-inflammatory Response to Acute Exacerbations of COPD? Lung. 2020;198(2):307-14.

18. Fadaei R, Dadmanesh M, Moradi N, Ahmadi R, Shokoohi Nahrkhalaji A, Aghajani H, et al. Serum levels of subfatin in patients with type 2 diabetes mellitus and its association with vascular adhesion molecules. Arch Physiol Biochem. 2020;126(4):335-40.

19. Gholamrezayi A, Mohamadinarab M, Rahbarinejad P, Fallah S, Barez SR, Setayesh L, et al. Characterization of the serum levels of Meteorin-like in patients with inflammatory bowel disease and its association with inflammatory cytokines. Lipids Health Dis. 2020;19(1):230.

20. Deniz R, Yavuzkir S, Ugur K, Ustebay DU, Baykus Y, Ustebay S, et al. Subfatin and asprosin, two new metabolic players of polycystic ovary syndrome. Journal of Obstetrics and Gynaecology. 2021;41(2):279-84.

21. Wu Q, Dan YL, He YS, Xiang K, Hu YQ, Zhao CN, et al. Circulating Meteorin-like Levels in Patients with Type 2 Diabetes Mellitus: A Meta-Analysis. Curr Pharm Des. 2020;26(44):5732-8.

22. Lappas M. Maternal obesity and gestational diabetes decrease Metrnl concentrations in cord plasma. J Matern Fetal Neonatal Med. 2021;34(18):2991-5.

23. Rupérez C, Ferrer-Curriu G, Cervera-Barea A, Florit L, Guitart-Mampel M, Garrabou G, et al. Meteorin-like/Meteorin-β protects heart against cardiac dysfunction. J Exp Med. 2021;218(5).

24. Schmid A, Karrasch T, Schäffler A. Meteorin-Like Protein (Metrnl) in Obesity, during Weight Loss and in Adipocyte Differentiation. J Clin Med. 2021;10(19).

25. Cherian P, Al-Khairi I, Jamal M, Al-Sabah S, Ali H, Dsouza C, et al. Association Between Factors Involved in Bone Remodeling (Osteoactivin and OPG) With Plasma Levels of Irisin and Meteorin-Like Protein in People With T2D and Obesity. Front Endocrinol (Lausanne). 2021;12:752892.

26. Grander C, Grabherr F, Enrich B, Meyer M, Mayr L, Schwärzler J, et al. Hepatic Meteorin-like and Krüppel-like Factor 3 are Associated with Weight Loss and Liver Injury. Exp Clin Endocrinol Diabetes. 2021.

27. Sobieh BH, Kassem DH, Zakaria ZM, El-Mesallamy HO. Potential emerging roles of the novel adipokines adipolin/CTRP12 and meteorin-like/METRNL in obesity-osteoarthritis interplay. Cytokine. 2021;138:155368.

28. Fouani FZ, Fadaei R, Moradi N, Zandieh Z, Ansaripour S, Yekaninejad MS, et al. Circulating levels of Meteorin-like protein in polycystic ovary syndrome: A case-control study. PLoS One. 2020;15(4):e0231943.

29. Tok Ö, Kişioğlu SV, Ersöz H, Kahveci B, Göktaş Z. Effects of increased physical activity and/or weight loss diet on serum myokine and adipokine levels in overweight adults with impaired glucose metabolism. J Diabetes Complications. 2021;35(5):107892.

30. Berghoff M, Hopfinger A, Rajendran R, Karrasch T, Schmid A, Schaffler A. Evidence of a Muscle-Brain Axis by Quantification of the Neurotrophic Myokine METRNL (Meteorin-Like Protein) in Human Cerebrospinal Fluid and Serum. Journal of Clinical Medicine. 2021;10(15).

31. Seyhanli ES, Guler O, Yasak IH, Koyuncu I. Investigation of the Correlation between Meteorin-Like Protein (Metrnl) and Thiol Balance in COVID-19 Patients. Clin Lab. 2021;67(12).

32. Ferns GA, Fekri K, Shahini Shams Abadi M, Banitalebi Dehkordi M, Arjmand MH. A meta-analysis of the relationship between serums metrnl-like protein/subfatin and risk of type 2 diabetes mellitus and coronary artery disease. Arch Physiol Biochem. 2021:1-7.

33. Cai J, Wang QM, Li JW, Xu F, Bu YL, Wang M, et al. Serum Meteorin-like is associated with weight loss in the elderly patients with chronic heart failure. J Cachexia Sarcopenia Muscle. 2022;13(1):409-17.

34. Ugur K, Erman F, Turkoglu S, Aydin Y, Aksoy A, Lale A, et al. Asprosin, visfatin and subfatin as new biomarkers of obesity and metabolic syndrome. Eur Rev Med Pharmacol Sci. 2022;26(6):2124-33.

35. Asprosin vasanbooamsT, M., Timurkaan ES. Two Important Players for Type 2 Diabetes Mellitus: Metrnl and Asprosin. Clin Lab. 2022;68(9).

36. Zhang S, Lei Y, Sun T, Gao Z, Li Z, Shen H. Elevated levels of Metrnl in rheumatoid arthritis: Association with disease activity. Cytokine. 2022;159:156026.

37. Ding X, Chang X, Wang J, Bian N, An Y, Wang G, et al. Serum Metrnl levels are decreased in subjects with overweight or obesity and are independently associated with adverse lipid profile. Front Endocrinol (Lausanne). 2022;13:938341.

38. Yilmaz M, Cagri Goktekin M, Ilhan N. Subfatin concentration decreases in acute coronary syndrome. Biochem Med (Zagreb). 2022;32(2):020704.

39. Timurkaan M, Timurkaan ES. Two Important Players for Type 2 Diabetes Mellitus: Metrnl and Asprosin. Clin Lab. 2022;68(9).
